# Evaluating calcineurin inhibitors as alternatives to steroids in treating Oral Lichen Planus: a systematic review and meta-analysis

**DOI:** 10.3389/froh.2026.1755525

**Published:** 2026-01-30

**Authors:** Bálint Zsombor Sárai, Ellay Gutmacher, Bianca Golzio Navarro Cavalcante, Kata Kelemen, Noémi Gede, Péter Hegyi, Dalma Tábi, Zsolt Németh, Zsolt M. Lohinai, Gábor Varga, Orsolya Németh

**Affiliations:** 1 Centre for Translational Medicine, Semmelweis University, Budapest, Hungary; 2 Department of Public Dental Health, Semmelweis University, Budapest, Hungary; 3 Department of Oral Biology, Semmelweis University, Budapest, Hungary; 4 Department of Prosthodontics, Semmelweis University, Budapest, Hungary; 5 Institute for Translational Medicine, Medical School, University of Pécs, Pécs, Hungary; 6 Institute of Pancreatic Diseases, Semmelweis University, Budapest, Hungary; 7 Department of Oro-maxillofacial Surgery and Stomatology, Semmelweis University, Budapest, Hungary; 8 Department of Restorative Dentistry and Endodontics, Semmelweis University, Budapest, Hungary

**Keywords:** burning sensation, morphology, network meta-analysis, pain, placebo

## Abstract

**Introduction:**

Oral Lichen Planus is a chronic inflammatory disease and a potentially malignant disorder of the oral mucosa. We aimed to investigate the possibility of replacing the gold-standard steroids (ST) due to their propensity for drug tolerance and numerous associated side effects. Recently, calcineurin inhibitors (CNI) have emerged as a promising alternative to ST. The main objective of the recent study was to compare the therapeutic effects of CNI and ST on OLP through pain severity, lesion morphology, and adverse effects.

**Materials and methods:**

A systematic search was conducted in 4 databases (Pubmed, Embase, Central, and Web of Science) on November 26th, 2023. Randomized controlled trials (RCTs) were selected for the analysis, which compared topically used CNIs, STs, or placebo. The outcomes were pain severity, oral mucosa morphology, and safety (adverse effects, especially transient burning). Odds ratios (ORs) and mean differences (MDs) with the random-effects model were calculated in the quantitative synthesis and interpreted with 95% confidence intervals (CIs).

**Results:**

A total of eleven RCTs were selected for quantitative analysis. Topically applied ST tended to decrease pain after four weeks of treatment (MD: −0.06, 95%CI: −0.55; 0.44) and promoted healing better (MD: −0.08, 95%CI: −0.74; 0.57) than measured in CNI therapy. Transient burning occurred more often in the CNI group (OR: 2.45, 95%CI: 0.55; 10.85).

**Discussion:**

Topical STs and topically used CNIs have a similar effect. However, data show a higher rate of side effects with CNI, particularly transient burning during the first two days of treatment.

**Clinical relevance:**

Topical STs and topically used CNIs have a similar effect. However, data show a higher rate of side effects with CNI, particularly transient burning during the first two days of treatment. Topically applied STs may remain the gold standard for symptomatic OLP. The evidence is currently insufficient to support replacing ST with CNI in OLP treatment in routine clinical practice.

**Systematic Review Registration:**

https://www.crd.york.ac.uk/PROSPERO/view/CRD42023486435, PROSPERO CRD42023486435.

## Introduction

Lichen Planus (LP) is an uncommon chronic disorder of unknown origin (1, 2). LP has different manifestations that affect the skin, oral cavity, genitalia, scalp, esophagus, or nails (3). A T-cell-mediated autoimmune mechanism has been proposed, especially CD8+ and CD4+ T-cells are directed against basal keratinocytes. The process may be enhanced by the overexpression and upregulation of intracellular adhesion molecule-1 (ICAM-1) and cytokines associated with a Th1 immune response (e.g., IFN-gamma, TNF-alpha, IL-1) (3, 4).

Oral involvement, called Oral Lichen Planus (OLP), is quite common and mainly affects the buccal mucosa, tongue, and gingiva, often with a bilateral and symmetrical appearance (2). The prevalence ranges from 0.5% to 2.2% in the population, and the cases mostly appear in women between the ages of 30 and 60 years (5, 6).

Although the risk is low, the condition is considered a potentially malignant disorder. Accurately assessing the transformation rate presents particular challenges, as independent studies have reported varying rates of malignant transformation. A review from 2007 concluded a prevalence of 0%–2% (7).

Regarding its appearance, literature distinguishes six types: reticular, papular, plaque-like, atrophic, erosive/ulcerative, and bullous (6, 8). In most cases, patients are asymptomatic and require no dedicated therapy, though mechanical damage can exacerbate the lesion.

The reticular type is the most commonly known type of OLP, and its appearance can be described as white interlacing white lines, the so-called Wickham striae. Reticular OLP is usually bilateral and requires no treatment (9). The atrophic and erosive types are painful and reduce the patient's quality of life; thus, maintenance of good oral hygiene and fabrication of suitable prostheses may play a significant role in the prevention and management of symptomatic OLP (7). The atrophic and erosive types are challenging to distinguish, and most cases are grouped together as atrophic-erosive types. These two present as atrophic and erythematous lesions, which may progress into ulceration. From that point, we can differentiate it as the ulcerative form of OLP (10). These types have to be treated because of the severe pain they cause during eating and maintaining oral hygiene.

While many drugs have been tested to manage the symptomatic form of OLP, including retinoids, steroids (ST), and photodynamic therapy, the success rate of these therapies has been unsteady (7). The gold standard is using topical/intralesional/systematic corticosteroids (betamethasone valerate, triamcinolone acetonide, fluocinolone acetonide, and clobetasol proprionate) (7).

Despite the therapeutical advances of ST used in the management of OLP, long-term follow-ups show an increase in disadvantages to consider (10). Although the treatment has several side effects, it must be repeated in every flare-up. Over time, adrenal suppression and secondary Candida infection have been reported by researchers, yet the weakening of the drug response can be the most problematic consequence to deal with (7, 10).

As the purpose is to reach an asymptomatic, high-quality life without any compromises, studies started to evaluate the use of topically administered calcineurin inhibitors (CNI) (cyclosporin, tacrolimus, pimecrolimus) based on the immune-mediated origin of OLP (11). CNIs are immunosuppressants primarily used during organ transplantations. The active agent of these drugs binds to different cytoplasmic proteins in the T-lymphocyte, inhibiting the activation of their response (11–13). Although clinical use confirmed the positive effects of CNI, there are many questions regarding their long-term effect not just on OLP, but on the human organs as well (11).

Our study aimed to investigate the advantages of replacing ST with CNI by comparing them for the reliable evaluation of CNI use in the treatment of symptomatic OLP, while also collecting data on any emerging side effects.

## Methods

We report our systematic review and network meta-analysis based on the recommendation of the PRISMA 2020 reporting guideline (14), while we also followed the Cochrane Handbook (15). The study protocol was registered on PROSPERO (CRD42023486435) with no deviations.

### Search strategy and eligibility criteria

The systematic search was conducted on November 26, 2023 in the following electronic databases: MEDLINE (via Pubmed), Embase, Cochrane Central Register of Controlled Trials (Central), and Clarivate (Web of Science), providing an extensive coverage of literature. Due to the time elapsed since the first systematic search, a new search was conducted on February 17, 2025. The following search key was used during both systematic searches in each database:

((“oral” AND “lichen” AND “planus”) OR OLP OR ORL)

AND

(tacrolimus OR clobetasol OR glucocortico* OR calcineurin OR steroid*)

To further ensure completeness, we also screened the reference lists of eligible studies manually. We used the following PICO framework: population (P): adult patients diagnosed with OLP; intervention (I): topical calcineurin inhibitors; control (C): topical steroids or placebo; outcome (O): severity of pain, lesion morphology, and adverse effects. No filters were applied during the search, but only English-language articles were considered eligible for this meta-analysis. We included only RCTs that reported data from the adult population (≥18 years of age) with the presence of histopathologically and clinically proven OLP and with the presence of symptoms (erosive, ulcerative, atrophic forms of OLP). Exclusion criteria were the following: any therapy for OLP three months before treatment period, pregnancy/breastfeeding in female patients, drug intolerance, and the presence of drug-induced lichenoid lesions. *In vitro* and animal studies, case reports, non-comparative studies, single-arm studies, studies with inappropriate comparator, cohort studies, and systematic reviews were excluded. The reference lists of eligible articles were also searched.

### Selection process

Two independent authors (EG and SB) performed the selection. For duplicate removal, we used a reference management software product (EndNote X9, Clarivate Analytics). After that, two independent reviewers (EG and SB) conducted the selection process by title, abstract, and full text following prediscussed aspects. Conflicts were resolved by a third, independent author (ON).

### Data collection process

Two reviewers (EG, SB) independently extracted all relevant data from the eligible articles into a predefined data table. Any disagreements were resolved by a third independent investigator (ON). The first authors’ names and publication years were extracted in all cases, and so were data on study design, intervention, the number of patients included, and their age, sex ratio, and inclusion/exclusion criteria used in the particular trial. Primary outcomes (the severity of pain using the Visual Analog Scale [VAS] (16)—Supplementary Figure S2, lesion morphology, using the Clinical Scoring system [CS] (17)—Supplementary Table S2, adverse effects) and any other additional outcomes were also collected by the reviewers. If there was a lack of eligible data, the authors of the eligible articles were contacted to provide further data. However, no data was available for us to include. Because of the lack of usable data, we had to group the follow-up periods close to similar, so weeks one and two and weeks three and four were pooled based on the healing time of the oral mucosa in the comparison of the investigated treatment regimens.

### Risk of bias assessment

The methodological quality was assessed by the two independent authors (EG, SB) separately for each outcome, using the RoB 2 tool (18) for RCTs by the Cochrane Collaboration. Bias was evaluated in five primary domains: randomization process, deviations from intended interventions, missing outcome data, measurement of the outcome, and selection of the reported result. Disagreements were resolved by the third, independent author (ON).

### Synthesis methods

#### Pairwise meta-analysis

Pairwise meta-analysis was carried out using R (*R Core Team 2021, v4.3.2, R: A Language and Environment for Statistical Computing. Vienna, Austria: R Foundation for Statistical Computing.* https://www.R-project.org/.*)* and the R package meta (version 6.5.0, 2. Schwarzer, Guido. 2022. Meta: General Package for Meta-Analysis. https://github.com/guido-s/meta/ https://link.springer.com/book/10.1007/978-3-319-21416-0). The mean values of the control group were subtracted from the mean values of the experimental group. To calculate study odds ratios and pooled odds ratios, the total number of patients and the number of those with the event of interest in each group were extracted or calculated from the studies where they were available. Random effect models were used to pool effect sizes. Between-study heterogeneity was described by the Higgins & Thompson's *I*^2^ statistics (https://onlinelibrary.wiley.com/doi/10.1002/sim.1186). The results were summarized on forest plots. Publication bias was not assessed due to the low number of included studies.

#### Network meta-analysis

Mean difference (MD) for continuous data Bayesian method was used to perform pairwise meta-analyses and network meta-analyses with the random effect model with 95% credible intervals (95% CrI). We optimized the model and generated posterior samples using the Monte-Carlo methods running in four chains. We set at least 20,000 adaptation iterations for convergence and 10,000 simulation iterations. We also ranked interventions by their posterior probability via calculating the surface under cumulative ranking (SUCRA) curve values. Plots were created to detect inconsistencies in outcomes. To examine inconsistency at the global level, the fit of the inconsistency model can be compared against a model in which consistency is assumed. Calculations were performed using the R package (version 4.1.1) and BUGSnet (version 1.1.0).

## Results

### Study selection

**Figure 1:**
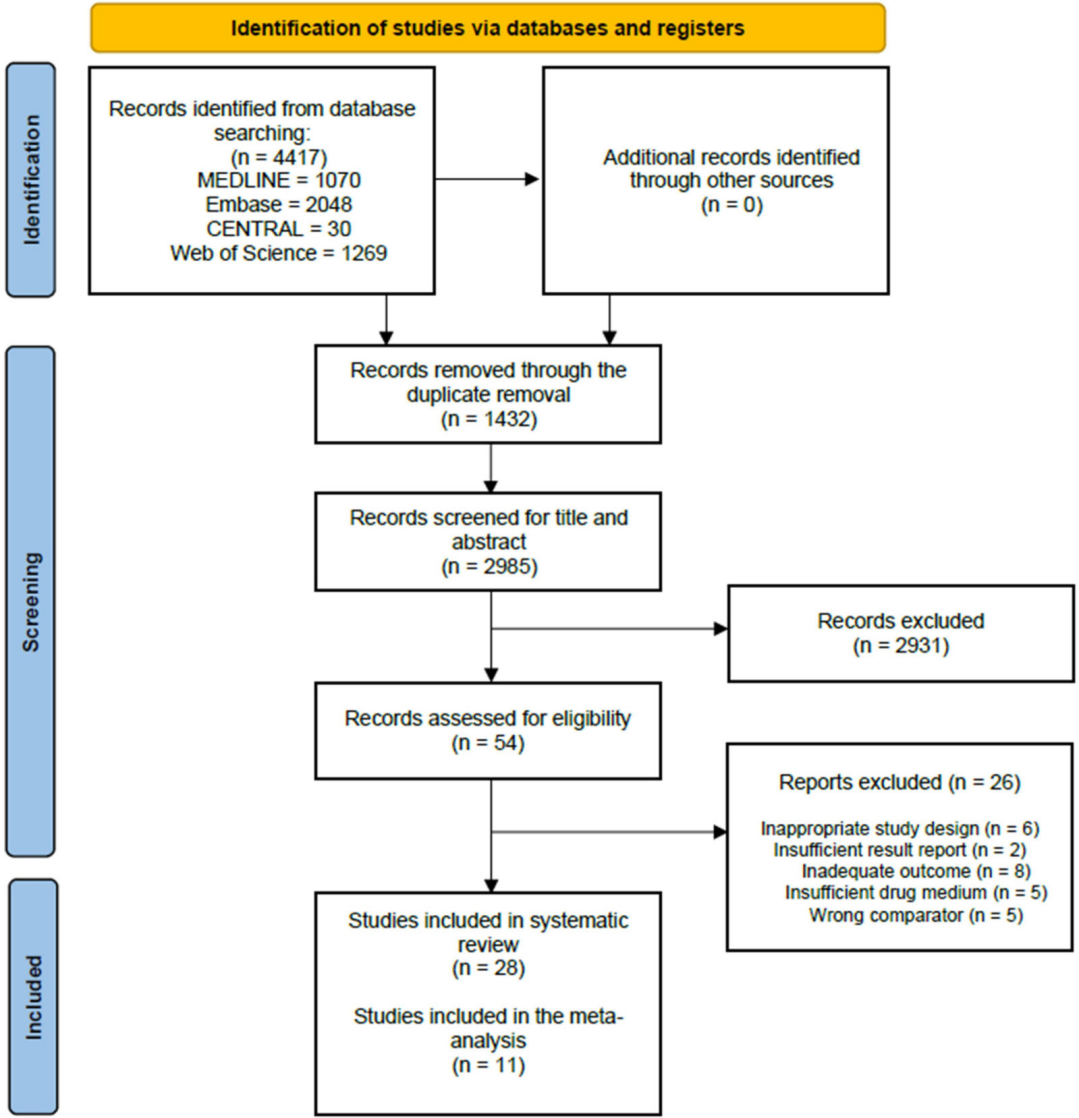
PRISMA flowchart showing the selection process.

We have identified 4,416 overall records in four databases. After removing duplicates, 2,948 studies were screened based on title and abstract, and 53 were eligible for full-text selection. Altogether, twenty-eight studies (1, 19–35) were finally selected for qualitative synthesis, and eleven studies (35–45) were eligible for quantitative synthesis. The selection process and Cohen's kappa values can be seen on the PRISMA flowchart (Figure 1).

### Study characteristics

#### Description of the included studies

The 28 included studies are characterized in Supplementary Table S1. All studies were randomized controlled trials in the quantitative analysis. All patients in the included studies were at least 18 years old. The selected RCTs were mainly two-arm studies, where different types of CNI (tacrolimus 0.1%, pimecrolimus 1%, cyclosporin 100 mg/ml) and ST (clobetasol propionate 0.05%, betamethasone 17-valerate 0.1%, triamcinolone acetonide 0.1%) were compared; in addition, one three-arm study was also included for the network meta-analysis part of our investigation, where placebo was used as a comparator. The treatment periods of the included studies ranged from 1 week to 12 weeks. Our primary outcomes were severity of pain (using the VAS), lesion morphology (using the Clinical Score), and transient burning (adverse effect).

#### Description of the excluded studies

Overall, 26 studies were excluded based on full-text during the selection process. Initially, six studies were excluded due to inappropriate study design (reviews, case reports, retrospective studies, and non-randomized studies were excluded). Among the remaining studies, five reports investigated insufficient drug medium (mouthwash, adhesive patch), and five studies used the wrong comparator (CNI vs. CNI, ST vs. ST, no comparator). Ten articles had both adequate study design and a sufficient comparator. However, two studies did not meet our requirements due to a lack of precise data reports, and eight used different outcome measurements during the investigation.

### Quantitative synthesis

Our meta-analysis included ten studies with 486 patients diagnosed with OLP. Three treatment regimens (CNI, ST, and placebo) were investigated regarding the drug response of OLP Patients.

### Severity of pain

**Figure 2:**
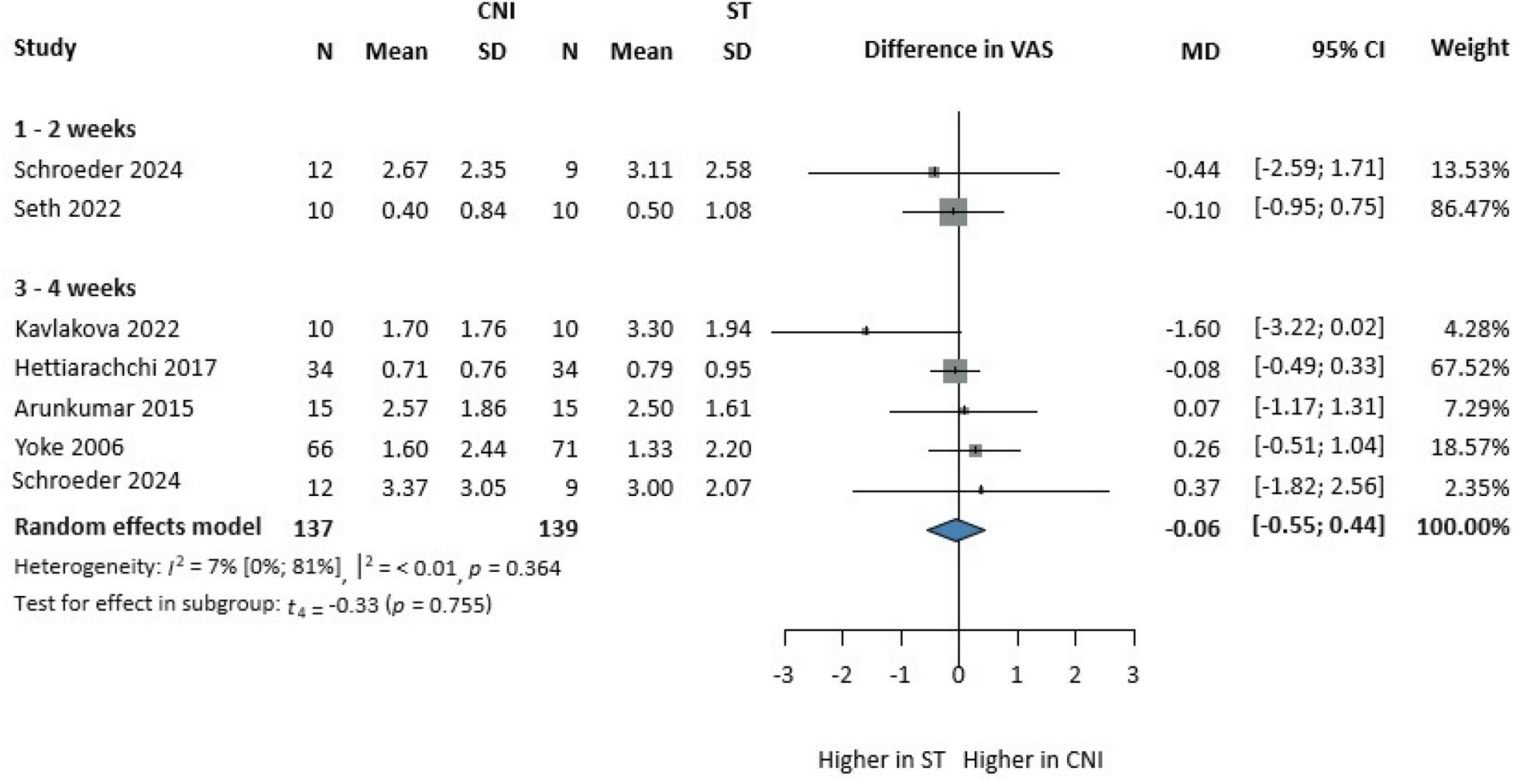
Comparison of CNI vs. ST on pain reduction using VAS change at 1-month follow-up.

The severity of pain was assessed with the help of the Visual Analog Scale (VAS) (0 = no pain; 10 = worst pain possible). Five articles (35, 36, 38, 43, 45) were included in the comparison, which used VAS to evaluate the pain before and after follow-up. After four weeks of treatment, there was no statistical and clinical difference between the groups on pain reduction (MD: −0.06, CI 95%: −0.55; 0.44). The heterogeneity was moderate [$I^{2}$: 7%, (0%; 81%)] (Figure 2).

**Figure 3:**
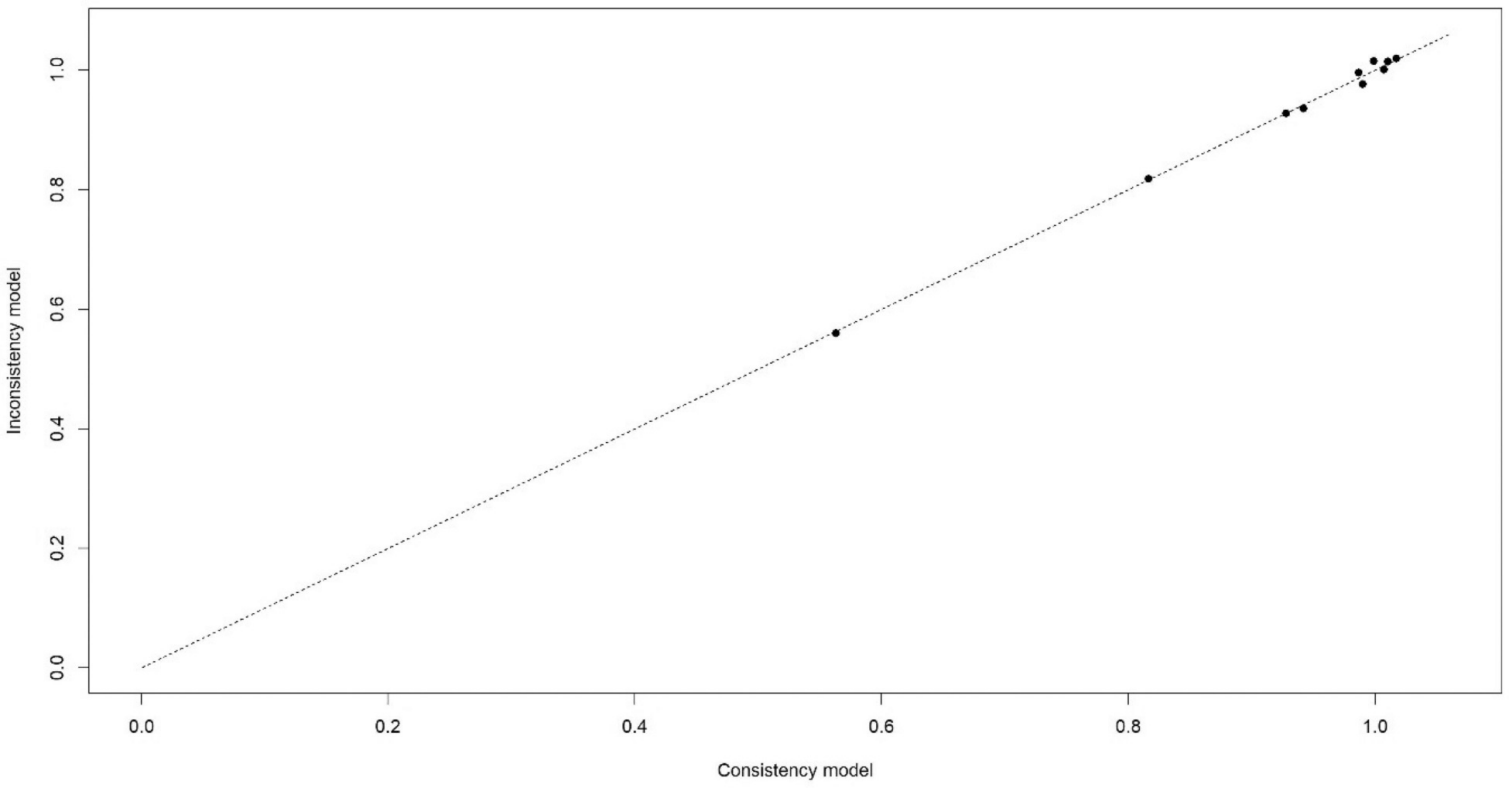
Consistency table of included data from five studies regarding VAS.

Five articles (36, 38, 41, 43, 45) were included in the network part of our meta-analysis, which included three different interventions: CNI, ST, and placebo. There was a direct comparison between CNI and ST, CNI and placebo, and an indirect comparison between ST and placebo with a total of 275 subjects (Supplementary Figure S1). The available data from the five selected studies were relatively homogeneous; our network meta-analysis demonstrated strong consistency (Figure 3).

**Figure 4:**
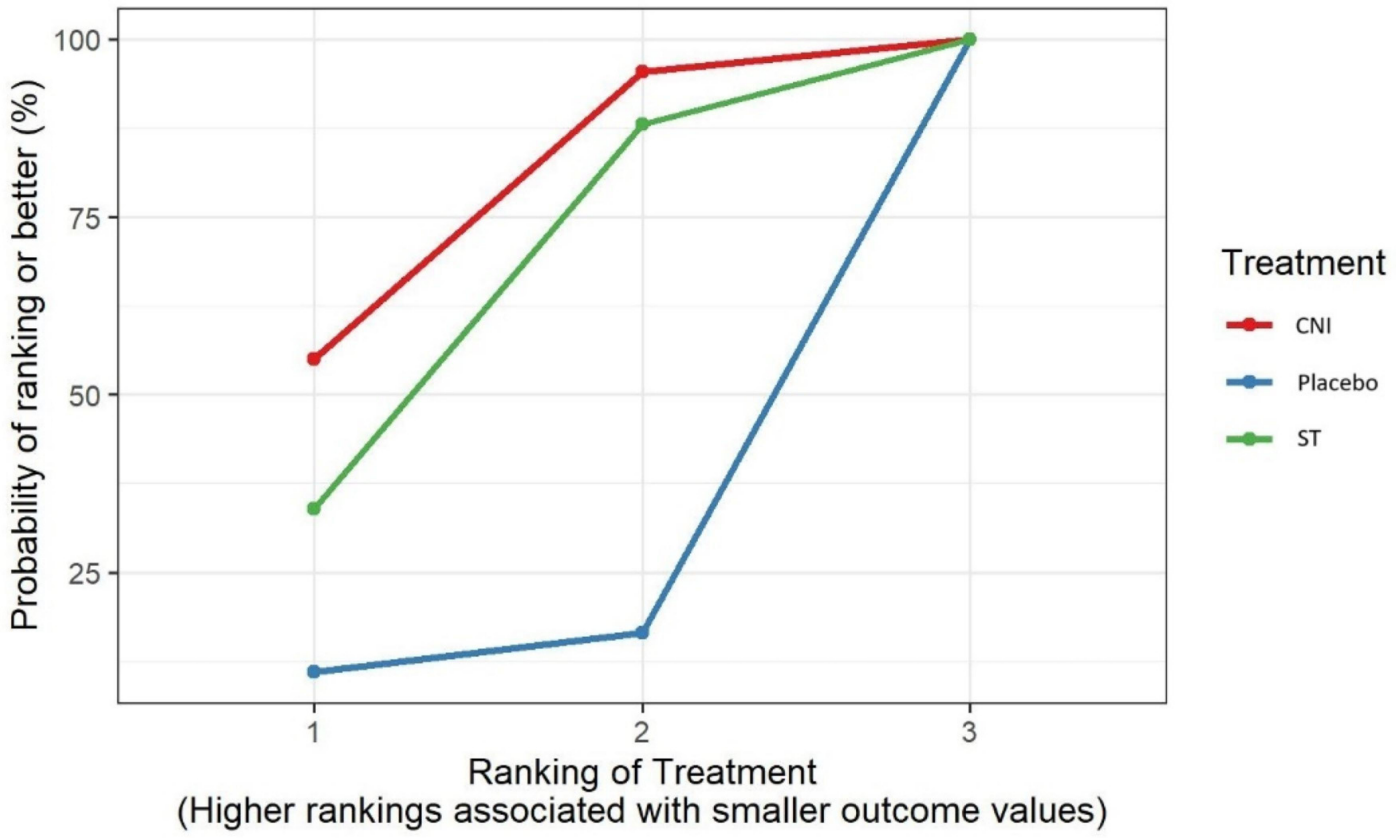
SUCRA values of CNI, ST, and placebo regarding VAS changes after four weeks of treatment.

The SUCRA values presented a superiority of CNI (highest rank at 75.27%), as the area under the curve was more extensive compared to ST (61%) and Placebo (13.74%) (Figure 4).

After four weeks of treatment, pain decreased more in the CNI group compared to the placebo and ST groups (MD: −1.32, CI: −3.57; 0.96) (MD: −0.11, CI: −0.97; 0.62), respectively. There was no statistically significant result based on moderate consistency (Table 1).

**Table 1:** Comparison of VAS changes after four weeks of treatment in CNI, ST, and placebo groups.

	Treatment			
Comparator		CNI	ST	Placebo
	CNI		0.11 (−0.62, 0.97)	1.32 (−0.96, 3.57)
	ST	−0.11 (−0.97, 0.62)		1.21 (−1.22, 3.56)
	Placebo	−1.32 (−3.57, 0.96)	−1.21 (−3.56, 1.22)	

### Lesion morphology

**Figure 5:**
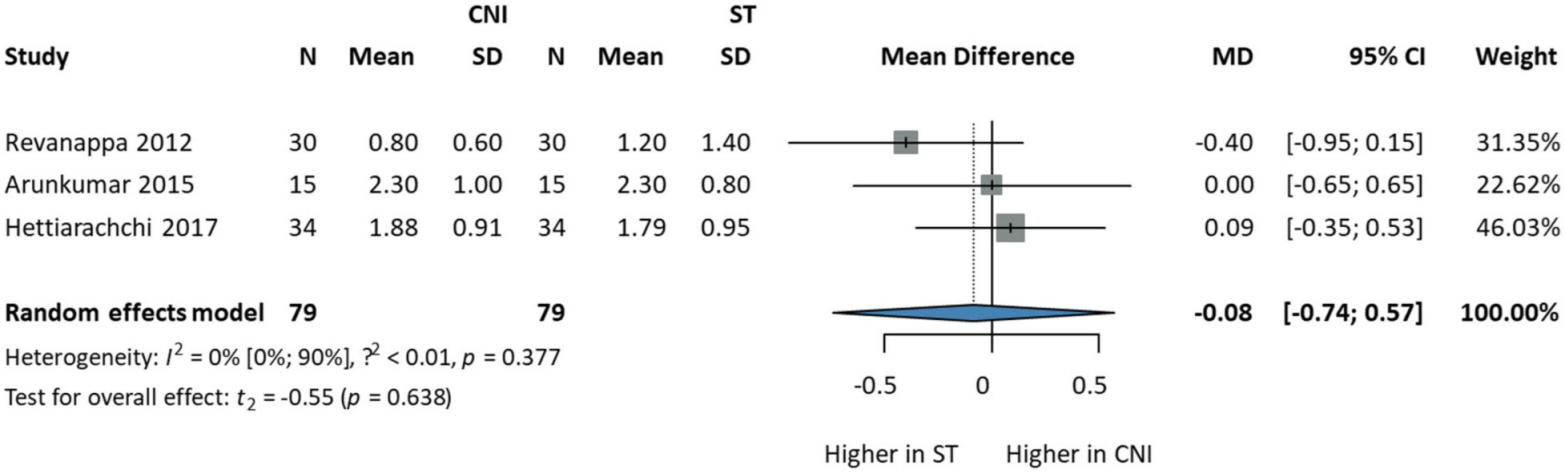
Comparison of CNI and ST for lesion morphology using CS changes after four weeks.

Lesion morphology was assessed using CS (Supplementary Table S2) according to Thongprasom et al. (16), which describes the mucosal morphology on a scale of 0−5. Three studies (36, 44, 45) comparing CNI and ST for lesion morphology, with 79 patients in each group, were included in the analysis. After four weeks of treatment, there was no statistically significant difference between the two groups (MD: −0.08, CI 95%: −0.74; 0.57). Heterogeneity was low [$I^{2}$: 0, (0%; 90%)] indicating consistent findings across studies. These results suggest that CNI and ST have comparable effects on lesion morphology over the treatment period (Figure 5).

### Adverse effects

**Figure 6:**
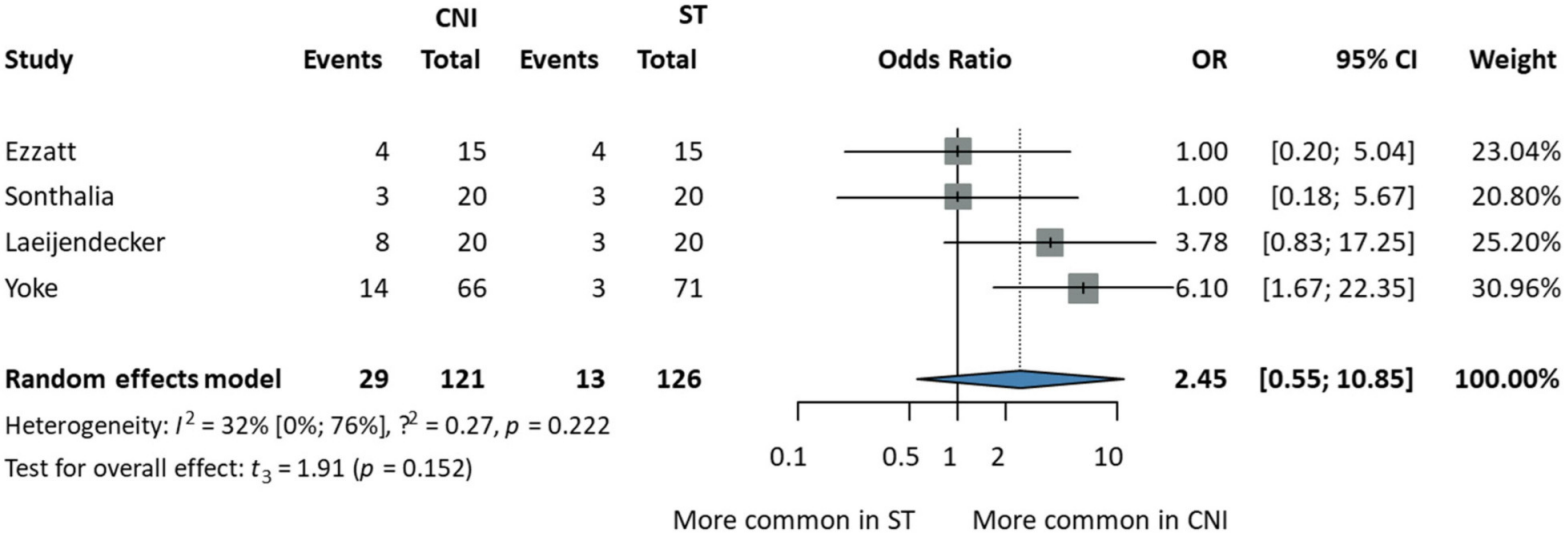
Comparison of transient burning occurrences in CNI and ST treatment regimens.

To evaluate the adverse effects of topically used drugs, a total of thirteen types of side effects were mentioned; nevertheless, only transient burning, which occurred during the first two days of the treatment period, had enough reports to be comparable. All other data is summed up in the systematic review. Transient burning was more common in the CNI group, but the difference was statistically insignificant yet clinically relevant (OR: 2.45, CI 95%: 0.55; 10.85). Heterogeneity was not very high [$I^{2}$: 32%, (0%; 76%)] (Figure 6).

The remaining extracted adverse effects indicate that neither ST nor CNI has a significant harmful impact on the human organism, as the most frequently reported adverse event was Oral Candidiasis, with 7 cases reported both in the CNI and ST groups (20, 40). The relatively low occurrence might be due to the prescribed antifungal mouthwash during the trials. Besides transient burning and Candida involvement, taste alteration (CNI = 4, ST = 1) and upper respiratory infections (CNI = 4, placebo = 4) were among the most frequently reported side effects. However, despite these findings, more research is required to better understand the short- and long-term disadvantages of using ST and CNI on OLP.

### Qualitative analysis

Seventeen studies could not be included in the meta-analysis due to inadequate outcome reports, lack of data, or insufficient outcome measurements. In six RCTs, different scales were used to evaluate mucosal appearance, which differed from CS and could not be used for the quantitative analysis [e.g., 0–4 (24), 0–3 (34), 1–12 (26), 0–130 (46)]. Relapse, burning sensation, and triggered pain were also inappropriate outcomes due to the lack of comparable studies. CS and VAS were reported in many articles, where we found a need for more essential data and accurate data presentation. Erythematous, Reticulation, and Ulceration Areas were measured in two articles (38, 41), based on which our findings are covered below.

### Pain severity

Ezzatt et al. presented the most significant improvement in each treatment regimen; however, they could not find a significance (37). While Georgaki et al. could report a significant decrease in pain scores in both groups (CNI and ST) after the treatment period, there was no statistical significance between ST and CNI VAS values. After five months of follow-up, they could see a trend in the CNI group for further improvement in VAS values (20). Neither Azizi et al. nor Kaur et al. could find a significant difference in VAS scores when comparing CNI to ST; however, Kaur et al. reported a lower rate of relapse in the CNI group after discontinuation of the therapy (CNI = 60%; ST = 75%) (1, 31). While Radfar et al. had the same result, they also observed a change in lesion sizes ($cm^{2}$) in all subjects. They could see a significant change in both treatment groups compared to baseline, but no difference (CNI = 82.6%; ST = 81.6% improvement) compared to each other after six weeks of treatment (25). Volz et al. and McCaughey et al. compared CNI to placebo regarding VAS scores. Volz et al. presented that CNI decreased both meal-triggered pain and continuous pain significantly during the study period, while placebo did not show such an improvement (22). McCaughey et al. not only reported similar VAS change results as previously observed but also noteda tendency for CNI to improve mucosal erythema more effectively than placebo (with no significance) (34). According to Malik et al., CNI therapy resulted in pain improvement in a higher percentage of the study population (93%) compared to ST (85%) (47). In terms of burning sensation and pain, Corrocher et al. could show a statistically significant difference between CNI and ST after four weeks of treatment period and two weeks of follow-up time, with a portion of patients in complete pain remission (23).

### Mucosal appearance

Ibrahim et al. investigated mucosal appearance based on CS but presented results on total atrophic area in $mm^{2}$ after eight weeks of treatment. Despite a positive tendency that CNI had shown after eight weeks of treatment, the overall improvement in lesion size of ST was better after twelve weeks of the study period, which was also statistically significant compared to CNI (33). Passeron et al. reported the superiority of CNI on CS (1–12) and Score of erosions (0–4) compared to placebo after four weeks of treatment (26).

### Erythematous area

We found two articles that measured the size of erythematous areas in $mm^{2}$. Yoke et al. reported a 56.27% improvement in the ST group after four weeks of treatment compared to the baseline value (38). Regarding CNI, Yoke et al. presented a 45.68% improvement, while Swift et al. presented a 66.99% improvement after the treatment period (38, 41). Placebo was the control for Swift et al., where the group showed a 17.95% worsening (41).

### Reticulation area

According to Yoke et al. ST therapy decreased the reticulation area by 47.19% in four weeks and 65.46% in eight weeks. With the same follow-ups, the change under the influence of CNI was 36.08% with a 48.52% improvement (38). A similar observation was performed in the reticulation area in the study of Swift et al., where the investigators compared CNI to placebo. After two weeks, CNI had a positive effect in decreasing the area by 48.11%; however, after four weeks, the investigators measured an 11.13% worsening. In the placebo group, a constant growth up to a 56.55% worsening was observed (41).

### Ulceration area

Yoke et al. and Swift et al. also presented data about the size and area of painful ulcers. According to Yoke, the mean size decreased by 72.70% from the baseline in the ST group and by 40.34% in the CNI group after four weeks (38), while Swift reported a 51.19% improvement in the CNI group and a 47.64% deterioration in the Placebo group (41). Their investigation resulted in an improvement in almost every regimen except when using a placebo.

### Risk of bias assessment

The results of the risk of bias assessment are shown in Supplementary Figure S3. Most of the studies had a moderate risk of bias (*n* = 19), primarily due to the selection of the reported results. In three cases, a high risk of bias occurred due to the randomization process, deviations from the intended intervention, and due to the selection of the reported results. The randomization process and deviations from the intended intervention caused concerns during the assessment.

In the outcome CS, which described the appearance of the oral mucosa after four weeks of treatment, three studies were included (36, 44, 45). All three studies had a moderate risk of bias because of their reported randomization process.

The overall risk of bias regarding the severity of pain (35, 36, 38, 43, 45) comparison is high due to the randomization process and the uncertain measurement of the VAS score.

### Quality of evidence

During the assessment of the evidence of our findings across pairwise meta-analysis, the GradePro tool was used in all three outcomes (VAS, CS, and Adverse effects). Investigating VAS and CS included a high overall risk of bias. However, we could reach a moderate certainty of evidence. In the case of Transient burning, the high certainty of evidence was assessed by the authors (Supplementary Figure S4) (48).

## Discussion

Symptomatic OLP decreases the patient's quality of life through constant discomfort, severe pain, and buccal tension for decades, not to mention the possibility of its malignant transformation. We investigated the effects of two different treatment regimens. Based on our results, CNI therapy is not superior compared to ST in the investigated outcomes. Our data provided significantly more information compared to the previously reported meta-analysis.

### Lesion morphology

Our meta-analysis showed no statistically significant difference between the CNI and ST groups in mucosal morphology. This is in line with the results of the included studies. Two articles (40, 46) were excluded from the quantitative analysis during the data extraction phase, which used different assessments to describe the morphology of the oral mucosa. Siponen et al. described no significant difference in lesion morphology (CS 0-130) changes between the two investigated drug groups after three weeks; however, three patients had no response when using ST on OLP (46). Sonthalia et al. (NCS) also reported no significance, but after two weeks of treatment, the authors anticipated a tendency of effectiveness differences between ST and CNI (40).

### Severity of pain

Our investigation did not find a statistically significant difference between CNI, ST, and Placebo in decreasing pain. Five articles (1, 20, 25, 31, 37) had to be excluded during the analysis because of a lack of data (no SD values presented). All five articles reported a significant decrease in VAS scores when focusing on ST. Despite minor differences in the reported responses of ST and CNI in pain measurement, we demonstrated a positive tendency when using CNI instead of ST.

### Adverse effects

Regarding Transient burning, a recent meta-analysis showed no statistically significant difference between CNI and ST. Adverse effects were reported orally by the patients in included RCTs. A previous meta-analysis by da Silva et al. identified transient burning as the most commonly reported adverse effect during CNI therapy. They also found out that dysgeusia (taste alteration), xerostomia, mucosal paresthesia, and sialorrhoea might be present during CNI therapy, which is in line with our results (49).

A possible additive effect for malignant transformation of OLP may be considered in the use of topically assigned CNIs and STs. A retrospective analysis from Bindakhil et al. indicated the potential impact of STs on delaying malignant transformation in OLP patients (50). Regarding CNIs, concerns have been raised regarding the potential carcinogenic risk of topical tacrolimus therapy. Becker et al. reported a hypothesis-generating case in which the development of cutaneous squamous cell carcinoma was temporally associated with long-term topical tacrolimus use, suggesting that carcinogenic effects beyond immune suppression could be possible (51).

An animal *in vivo* study has been conducted by Li et al., where the authors investigated the effects of intraperitoneally administered Tacrolimus on OSCC (Oral Squamous Cell Carcinoma) progression and tumor formation. Based on their findings, Tacrolimus inhibited the malignant transformation of oral cell dysplasia to OSCC and inhibited tumor formation in rats (52). Future research focusing on the influence of CNIs’ effect on carcinogenesis in OLP subjects could address existing knowledge gaps and facilitate a thorough evaluation of their safety.

### Description of previous analyses

In a previous meta-analysis by da Silva et al., clinical response and symptom resolution of CNIs were compared to ST and placebo (49). Their findings were similar to ours, as they couldn't find any superiority between CNIs separately (tacrolimus, pimecrolimus, and cyclosporin) and pooled corticosteroids in clinical response or symptom resolution. However, in their analysis, pimecrolimus showed superiority compared to Placebo in clinical response. They also reported the most frequent side effect, transient burning, which correlates with our findings (49).

Another meta-analysis from India with 11 included RCTs also investigated the possible use of tacrolimus instead of steroids (53). Tacrolimus was mainly compared to pimecrolimus, clobetasol propionate, and triamcinolone acetonide. Neither triamcinolone acetonide nor clobetasol showed superior efficacy compared to tacrolimus in clinical response and lesion size regression (53).

Leong and colleagues conducted a similar network meta-analysis in 2023, during which nine different treatment methods were investigated (54). Regarding Clinical Improvement, the authors presented a significant change in CNIs and STs compared to Placebo, however, there was no statistically significant difference between CNIs and STs. Regarding Pain scores, CNIs and STs tended to decrease pain compared to Placebo, with no statistical significance. Similar to a recent meta-analysis, Transient burning was the most common adverse effect (54). Sun et al. have also reported a statistically insignificant difference between CNIs and STs in terms of pain severity, and mucosal morphology, similar to a recent analysis (55).

During the outcome analysis, difficulties arose because of the heterogeneity of the outcome measures among the included RCTs (49, 53). During our analysis, we performed a more accurate and transparent analysis, where different outcome measures (pain severity, lesion morphology, and adverse effects) were investigated separately rather than pooling heterogeneous endpoints to result in a higher level of evidence. To support our results, both pairwise and network meta-analyses were performed, with more recent RCTs included. To assess the certainty of evidence, GRADE was applied during our work. Based on the above, our findings are in agreement with the previous publications (49, 53).

### Strengths and limitations

We implemented a rigorous methodology, and only those randomized controlled trials were included in our meta-analysis where we could use direct and indirect comparisons. The selected articles had a low to moderate bias risk, resulting from the imprecisely presented confounding factors.

Our meta-analysis has some limitations. Due to the inadequate data (not standardized outcome measurements), we analyzed small sample sizes from heterogeneous populations. Further research with standardized outcome measurements is necessary to acquire a large amount of data for more reliable evidence. Unfortunately, the lack of follow-up data suggests further research to investigate long-term effects and safety. Also, we could only use the data where the mean and standard deviation values were reported accurately. Additionally, only English-language articles were selected for the recent analysis.

### Implication for practice

Our findings indicate that CNIs and STs are comparable in symptomatic OLP, but do not demonstrate superior efficacy. As scientific results need rapid clinical translation (56, 57), clinicians should continue considering ST as the gold standard for OLP treatment, considering its proven efficacy and safety. However, given the comparable outcomes between ST and CNI, CNI may be a valid alternative for replacing STs in cases where steroid resistance or intolerance is involved.

### Implication for research

Future research should focus on extended follow-up periods to evaluate long-term efficacy and potential adverse treatment events for OLP. We emphasize the importance of further research on topical CNI therapy regarding the influence of malignant transformation of OLP. Additionally, we recommend developing or adopting a universally accepted scoring system for assessing the mucosal lesion and facilitating its incorporation into everyday practice.

## Conclusion

Topical CNIs demonstrate a pain-relieving and mucosal healing efficacy similar to topical STs in OLP, however, SUCRA rankings show a directional trend favouring CNIs. It is important to note that CNIs are associated with a higher incidence of adverse events during the initial two days after treatment. As such, current evidence does not support the replacement of gold-standard STs with CNIs in routine clinical practice, except in cases where patients exhibit resistance to STs.

## Data Availability

The original contributions presented in the study are included in the article/Supplementary Material, further inquiries can be directed to the corresponding author.

## References

[1] Azizi A Lawaf S. The comparison of efficacy of adcortyl ointment and topical tacrolimus in treatment of erosive oral lichen planus. J Dent Res Dent Clin Dent Prospects. (2007) 1(3):99–102. 10.5681/joddd.2007.017 23277842 PMC3529890

[2] Nuzzolo P Celentano A Bucci P Adamo D Ruoppo E Leuci S Lichen planus of the lips: an intermediate disease between the skin and mucosa? Retrospective clinical study and review of the literature. Int J Dermatol. (2016) 55(9):e473–81. 10.1111/ijd.13265 26992292

[3] Beth G Goldstein M Goldstein AO Mostow E. Lichen Planus (2024). Available online at: https://www.uptodate.com/contents/lichen-planus (Accessed September 02, 2024).

[4] Lehman JS Tollefson MM Gibson LE. Lichen planus. Int J Dermatol. (2009) 48(7):682–94. 10.1111/j.1365-4632.2009.04062.x 19570072

[5] Al-Hashimi I Schifter M Lockhart PB Wray D Brennan M Migliorati CA Oral lichen planus and oral lichenoid lesions: diagnostic and therapeutic considerations. Oral Surg Oral Med Oral Pathol Oral Radiol Endod. (2007) 103(Suppl):S25.e1–12. 10.1016/j.tripleo.2006.11.001 17261375

[6] McCartan BE Healy CM. The reported prevalence of oral lichen planus: a review and critique. J Oral Pathol Med. (2008) 37(8):447–53. 10.1111/j.1600-0714.2008.00662.x 18624932

[7] Ismail SB Kumar SK Zain RB. Oral lichen planus and lichenoid reactions: etiopathogenesis, diagnosis, management and malignant transformation. J Oral Sci. (2007) 49(2):89–106. 10.2334/josnusd.49.89 17634721

[8] Roopashree MR Gondhalekar RV Shashikanth MC George J Thippeswamy SH Shukla A. Pathogenesis of oral lichen planus—a review. J Oral Pathol Med. (2010) 39(10):729–34. 10.1111/j.1600-0714.2010.00946.x 20923445

[9] Chiang CP Yu-Fong Chang J Wang YP Wu YH Lu SY Sun A. Oral lichen planus—differential diagnoses, serum autoantibodies, hematinic deficiencies, and management. J Formos Med Assoc. (2018) 117(9):756–65. 10.1016/j.jfma.2018.01.021 29472048

[10] Akram Z Abduljabbar T Vohra F Javed F. Efficacy of low-level laser therapy compared to steroid therapy in the treatment of oral lichen planus: a systematic review. J Oral Pathol Med. (2018) 47(1):11–7. 10.1111/jop.12619 28766756

[11] Al Johani KA Hegarty AM Porter SR Fedele S. Calcineurin inhibitors in oral medicine. J Am Acad Dermatol. (2009) 61(5):829–40. 10.1016/j.jaad.2009.03.012 19836643

[12] Liu ZW Lin TN He GZ. [Research of compound cyclosporin A mouthwash in the treatment of oral lichen planus]. Hunan Yi Ke Da Xue Xue Bao. (2000) 25(2):183–4. 12212218

[13] de Paulis A Stellato C Cirillo R Ciccarelli A Oriente A Marone G. Anti-inflammatory effect of FK-506 on human skin mast cells. J Invest Dermatol. (1992) 99(6):723–8. 10.1111/1523-1747.ep12614216 1281861

[14] Page MJ McKenzie JE Bossuyt PM Boutron I Hoffmann TC Mulrow CD The PRISMA 2020 statement: an updated guideline for reporting systematic reviews. Br Med J. (2021) 372:n71. 10.1136/bmj.n71 33782057 PMC8005924

[15] Higgins JPTJ Chandler J Cumpston M Li T Page MJ Welch VA. Cochrane Handbook for Systematic Reviews of Interventions Version 6.1. London: Cochrane (2020).

[16] Visual Analog Scale. Available online at: https://rebelem.com/haloperidol-for-treatment-of-headache-in-the-emergency-department/haldol-for-headache-vas-score/ (Accessed November 27, 2024).

[17] Thongprasom K Luangjarmekorn L Sererat T Taweesap W. Relative efficacy of fluocinolone acetonide compared with triamcinolone acetonide in treatment of oral lichen planus. J Oral Pathol Med. (1992) 21(10):456–8. 10.1111/j.1600-0714.1992.tb00974.x 1460584

[18] Sterne JAC Savović J Page MJ Elbers RG Blencowe NS Boutron I Rob 2: a revised tool for assessing risk of bias in randomised trials. Br Med J. (2019) 366:l4898. 10.1136/bmj.l4898 31462531

[19] Arduino PG Campolongo MG Sciannameo V Conrotto D Gambino A Cabras M Randomized, placebo-controlled, double-blind trial of clobetasol propionate 0.05% in the treatment of oral lichen planus. Oral Dis. (2018) 24(5):772–7. 10.1111/odi.12821 29297958

[20] Georgaki M Piperi E Theofilou VI Pettas E Stoufi E Nikitakis NG. A randomized clinical trial of topical dexamethasone vs. Cyclosporine treatment for oral lichen planus. Med Oral Patol Oral Cir Bucal. (2022) 27(2):e113–e24. 10.4317/medoral.25040 34564686 PMC8898582

[21] Voûte AB Schulten EA Langendijk PN Kostense PJ van der Waal I. Fluocinonide in an adhesive base for treatment of oral lichen planus. A double-blind, placebo-controlled clinical study. Oral Surg Oral Med Oral Pathol. (1993) 75(2):181–5. 10.1016/0030-4220(93)90091-H 8426717

[22] Volz T Caroli U Lüdtke H Bräutigam M Kohler-Späth H Röcken M Pimecrolimus cream 1% in erosive oral lichen planus—a prospective randomized double-blind vehicle-controlled study. Br J Dermatol. (2008) 159(4):936–41. 10.1111/j.1365-2133.2008.08726.x 18647310

[23] Corrocher G Di Lorenzo G Martinelli N Mansueto P Biasi D Nocini PF Comparative effect of tacrolimus 0.1% ointment and clobetasol 0.05% ointment in patients with oral lichen planus. J Clin Periodontol. (2008) 35(3):244–9. 10.1111/j.1600-051X.2007.01191.x 18269664

[24] Rodstrom PO Hakeberg M Jontell M Nordin P. Erosive oral lichen planus treated with clobetasol propionate and triamcinolone acetonide in orabase: a double-blind clinical trial. J Dermatol Treatment. (1994) 5(1):7–10. 10.3109/09546639409081837

[25] Radfar L Wild RC Suresh L. A comparative treatment study of topical tacrolimus and clobetasol in oral lichen planus. Oral Surg Oral Med Oral Pathol Oral Radiol Endod. (2008) 105(2):187–93. 10.1016/j.tripleo.2007.07.029 18230389

[26] Passeron T Lacour JP Fontas E Ortonne JP. Treatment of oral erosive lichen planus with 1% pimecrolimus cream: a double-blind, randomized, prospective trial with measurement of pimecrolimus levels in the blood. Arch Dermatol. (2007) 143(4):472–6. 10.1001/archderm.143.4.472 17438179

[27] Gorouhi F Solhpour A Beitollahi JM Afshar S Davari P Hashemi P Randomized trial of pimecrolimus cream versus triamcinolone acetonide paste in the treatment of oral lichen planus. J Am Acad Dermatol. (2007) 57(5):806–13. 10.1016/j.jaad.2007.06.022 17658663

[28] Arduino PG Carbone M Della Ferrera F Elia A Conrotto D Gambino A Pimecrolimus vs. tacrolimus for the topical treatment of unresponsive oral erosive lichen planus: a 8 week randomized double-blind controlled study. J Eur Acad Dermatol Venereol. (2014) 28(4):475–82. 10.1111/jdv.12128 23451852

[29] Sivaraman S Santham K Nelson A Laliytha B Azhalvel P Deepak J. A randomized triple-blind clinical trial to compare the effectiveness of topical triamcinolone acetonate (0.1%), clobetasol propionate (0.05%), and tacrolimus orabase (0.03%) in the management of oral lichen planus. J Pharmacy Bioallied Sci. (2016) 8:S86–S9. 10.4103/0975-7406.191976 PMC5074049 27829754

[30] Siponen M Huuskonen L Kallio-Pulkkinen S Nieminen P Salo T. Tacrolimus and triamcinolone acetonide in OLP: a placebo-controlled RCT. Oral Dis. (2016) 22:24. 10.1111/odi.12559 28168769

[31] Kaur M Kathariya R Bontha SC Chavva SC Krishna MB. Topical clobetasol (0.025%) and tacrolimus (0.1%) in the management of oral lichen planus: a comparative study. Res J Pharm Biol Chem Sci. (2016) 7(6):2492–9.

[32] Malik S Hassan F Haq UUL Jabeen W Farooq Kayani SG. Topical tacrolimus vs topical triamcinolone equally effective in the treatment of oral lichen planus. Pak J Med Health Sci. (2021) 15(7):1500–2. 10.53350/pjmhs211571500

[33] Ibrahim SS Ragy NI Nagy NA El-Kammar H Elbakry AM Ezzatt OM. Evaluation of muco-adhesive tacrolimus patch on caspase-3 induced apoptosis in oral lichen planus: a randomized clinical trial. BMC oral Health. (2023) 23(1):99. 10.1186/s12903-023-02803-8 36788511 PMC9930326

[34] McCaughey C Machan M Bennett R Zone JJ Hull CM. Pimecrolimus 1% cream for oral erosive lichen planus: a 6-week randomized, double-blind, vehicle-controlled study with a 6-week open-label extension to assess efficacy and safety. J Eur Acad Dermatol Venereol. (2011) 25(9):1061–7. 10.1111/j.1468-3083.2010.03923.x 21175873

[35] Schroeder FMM Pedraça ES Palma VM Carrard VC Martins MAT Maito F Topical tacrolimus orabase versus topical clobetasol propionate orabase in the treatment of symptomatic oral lichen planus: a pilot randomized study. Clin Oral Investig. (2024) 28(10):559. 10.1007/s00784-024-05943-5 39348002

[36] Hettiarachchi PVKS Hettiarachchi RM Jayasinghe RD Sitheeque M. Comparison of topical tacrolimus and clobetasol in the management of symptomatic oral lichen planus: a double-blinded, randomized clinical trial in Sri Lanka. J Investig Clin Dent. (2017) 8(4):1166–76. 10.1111/jicd.12237 27633647

[37] Ezzatt OM Helmy IM. Topical pimecrolimus versus betamethasone for oral lichen planus: a randomized clinical trial. Clin Oral Investig. (2019) 23(2):947–56. 10.1007/s00784-018-2519-6 29909565

[38] Yoke PC Tin GB Kim MJ Rajaseharan A Ahmed S Thongprasom K A randomized controlled trial to compare steroid with cyclosporine for the topical treatment of oral lichen planus. Oral surgery, oral medicine, oral pathology. Oral Radiol Endodontics. (2006) 102(1):47–55. 10.1016/j.tripleo.2005.09.006 16831672

[39] Seth V Patil R Moger G Singh U Sharma A Saxena S. Comparative evaluation of the efficacy of topical amlexanox 5%, triamcinolone acetonide 0.1%, and tacrolimus 0.03% in the treatment of oral erosive lichen Planus—a double-blinded randomized clinical trial. J Indian Acad Oral Med Radiol. (2022) 34(2):136–40. 10.4103/jiaomr.jiaomr_16_21

[40] Sonthalia S Singal A. Comparative efficacy of tacrolimus 0.1% ointment and clobetasol propionate 0.05% ointment in oral lichen planus: a randomized double-blind trial. Int J Dermatol. (2012) 51(11):1371–8. 10.1111/j.1365-4632.2012.05459.x 23067089

[41] Swift JC Rees TD Plemons JM Hallmon WW Wright JC. The effectiveness of 1% pimecrolimus cream in the treatment of oral erosive lichen planus. J Periodontol. (2005) 76(4):627–35. 10.1902/jop.2005.76.4.627 15857105

[42] Laeijendecker R Tank B Dekker SK Neumann HAM. A comparison of treatment of oral lichen planus with topical tacrolimus and triamcinolone acetonide ointment. Acta Derm Venereol. (2006) 86(3):227–9. 10.2340/00015555-0070 16710580

[43] Kavlakova L. Evaluation of clinical efficacy of topical tacrolimus 0.1% and clobetasol propionate 0.05% in desquamative gingivitis, manifestation of oral lichen planus. Folia Med (Plovdiv). (2022) 64(3):415–21. 10.3897/folmed.64.e62851 35856102

[44] Revanappa MM Naikmasur VG Sattur AP Kailasam S. Evaluation of efficacy of tacrolimus 0.1% in orabase and triamcinolone acetonide 0.1% in orabase in the management of symptomatic oral lichen planus randomized single blind control study. J Indian Acad Oral Med Radiol. (2012) 24:269–73. 10.5005/jp-journals-10011-1311

[45] Arunkumar S Kalappanavar AN Annigeri RG Kalappa SG. Relative efficacy of pimecrolimus cream and triamcinolone acetonide paste in the treatment of symptomatic oral lichen planus. Indian J Dent. (2015) 6(1):14–9. 10.4103/0975-962X.151692 25767355 PMC4357072

[46] Siponen M Huuskonen L Kallio-Pulkkinen S Nieminen P Salo T. Topical tacrolimus, triamcinolone acetonide, and placebo in oral lichen planus: a pilot randomized controlled trial. Oral Dis. (2017) 23(5):660–8. 10.1111/odi.12653 28168769

[47] Malik U Gupta S Malik SD Vashishth S Zaheeruddin Raju MS. Treatment of symptomatic oral lichen planus (OLP) with 0.1% tacrolimus powder in oraguard-B—a pilot prospective study. Saudi Dent J. (2012) 24(3):143–8. 10.1016/j.sdentj.2012.05.002 23960543 PMC3729288

[48] Schünemann HBJ Guyatt G Oxman A. GRADE handbook for Grading Quality of Evidence and Strength of Recommendations. Hamilton, ON: The GRADE Working Group (2013).

[49] da Silva EL de Lima TB Rados PV Visioli F. Efficacy of topical non-steroidal immunomodulators in the treatment of oral lichen planus: a systematic review and meta-analysis. Clin Oral Investig. (2021) 25(9):5149–69. 10.1007/s00784-021-04072-7 34342763

[50] Bindakhil M Akintoye S Corby P Stoopler ET Greenberg MS Shanti R Influence of topical corticosteroids on malignant transformation of oral lichen planus. J Oral Pathol Med. (2022) 51(2):188–93. 10.1111/jop.13257 34748663

[51] Becker JC Houben R Vetter CS Bröcker EB. The carcinogenic potential of tacrolimus ointment beyond immune suppression: a hypothesis creating case report. BMC Cancer. (2006) 6(7):1–7. 10.1186/1471-2407-6-7 16405733 PMC1386691

[52] Li Y Wang Y Li J Ling Z Chen W Zhang L Tacrolimus inhibits oral carcinogenesis through cell cycle control. Biomed Pharmacother. (2021) 139:111545. 10.1016/j.biopha.2021.111545 33873145

[53] Pinto J Waghmare M Bhor K Santosh V Manoj R Samson S. Efficacy and safety of topical tacrolimus in comparison with topical corticosteroids, calcineurin inhibitors, retinoids and placebo in oral lichen Planus: an updated systematic review and meta-analysis. Asian Pac J Cancer Prev. (2023) 24(2):389–400. 10.31557/APJCP.2023.24.2.389 36853285 PMC10162640

[54] Leong XY Gopinath D Syeed SM Veettil SK Shetty NY Menon RK. Comparative efficacy and safety of interventions for the treatment of oral lichen Planus: a systematic review and network meta-analysis. J Clin Med. (2023) 12(8):2763. 10.3390/jcm12082763 37109100 PMC10144824

[55] Sun SL Liu JJ Zhong B Wang JK Jin X Xu H Topical calcineurin inhibitors in the treatment of oral lichen planus: a systematic review and meta-analysis. Br J Dermatol. (2019) 181(6):1166–76. 10.1111/bjd.17898 30903622

[56] Hegyi P Erőss B Izbéki F Párniczky A Szentesi A. Accelerating the translational medicine cycle: the academia Europaea pilot. Nat Med. (2021) 27(8):1317–9. 10.1038/s41591-021-01458-8 34312557

[57] Hegyi P Petersen OH Holgate S Erőss B Garami A Szakács Z Academia Europaea position paper on translational medicine: the cycle model for translating scientific results into community benefits. J Clin Med. (2020) 9(5):1532. 10.3390/jcm9051532 32438747 PMC7290380

