# Supplementary material for: Evaluating calcineurin inhibitors as alternatives to steroids in treating Oral Lichen Planus: a systematic review and meta-analysis
Source: Front Oral Health. 2026 Jan 30;7:1755525. doi: 10.3389/froh.2026.1755525 (PMC12901443; doi:10.3389/froh.2026.1755525)
Supplement: Supplementary file 1 [file Supplementaryfile1.docx]

**SUPPLEMENTARY MATERIAL**

| **Author (year)** | **Number of analyzed patients (female %)** | **Mean age - Intervention** | **Mean age - Control** | **Measurements**  **(week)** | **Drug application/day** | **Intervention** | **Control** | **Outcomes** |
| --- | --- | --- | --- | --- | --- | --- | --- | --- |
| ***Hettiarachchi, P. et al. (2017)*** | **68 (63.24)** | **46.65** | **46.88** | **0, 4, 8, 12** | **NA** | **CNI**  **(Tacrolimus 0.1%)** | **ST**  **(Clobetasol propionate 0.05%)** | **CS**  **VAS** |
| ***Ezzatt, O. M. et al. (2019)*** | **30 (73.34)** | **49.03** | **50.75** | **0, 1, 2, 4** | **NA** | **CNI**  **(Pimecrolimus 1%)** | **ST**  **(Betamethasone 17-valerate 0.1%)** | **CS**  **VAS** |
| ***Yoke, P. C. et al. (2006)*** | **137 (68.61)** | **43.90** | **43.50** | **0, 2, 4, 8** | **3** | **ST**  **(NA)** | **CNI**  **(Cyclosporin)** | **CS**  **VAS**  **Erythematous area**  **Reticulation area**  **Ulceration area** |
| ***Seth, V. et al. (2022)*** | **30 (NA)** | **1: NA**  **2: NA** | **NA** | **0, 1, 2** | **3** | **1: ST**  **(Triamcinolone acetonide 0.1%)**  **2: CNI**  **(Tacrolimus 0.03%)** | **Placebo** | **VAS**  **Erosive area** |
| ***Sonthalia, S. et al. (2012)*** | **40 (60.00)** | **34.35** | **35.05** | **0, 2, 4, 8** | **2** | **ST**  **(Clobetasol propionate 0.05%)** | **CNI**  **(Tacrolimus 0.1%)** | **NCS** |
| ***Swift, J. C. et al. (2005)*** | **20 (NA)** | **NA** | **NA** | **0, 2, 4** | **2** | **CNI**  **(Pimecrolimus 1%)** | **Placebo** | **VAS**  **Erythematous area**  **Reticulation area**  **Ulceration area** |
| ***Laeijendecker, R. et al. (2006)*** | **40 (75.00)** | **57.00** | **58.00** | **0, 6** | **4** | **CNI**  **(Tacrolimus 0.1%)** | **ST**  **(Triamcinolone acetonide 0.1%)** | **Improvement (ordinal scale)** |
| ***Kavlakova, L. et al. (2022)*** | **20 (90.00)** | **NA** | **NA** | **0, 3** | **2** | **ST**  **(Clobetasol propionate 0.05%)** | **CNI**  **(Tacrolimus 0.1%)** | **VAS**  **Relapse** |
| ***Revanappa, M. M. et al. (2012)*** | **60 (38.34)** | **42.60** | **48.40** | **0, 1, 2** | **3** | **CNI**  **(Tacrolimus 0.1%)** | **ST**  **(Triamcinolone acetonide 0.1%)** | **CS**  **VAS** |
| ***Arunkumar, S. et al. (2015)*** | **30 (NA)** | **NA** | **NA** | **0, 4, 8** | **4** | **CNI**  **(Pimecrolimus 1%)** | **ST**  **(Triamcinolone acetonide 0.1%)** | **CS**  **VAS** |
| ***Schroeder, F. M. M. et al.*** | **21 (95)** | **60.7** | **64.5** | **0, 2, 4** | **4** | **CNI**  **(Tacrolimus 0.1%)** | **ST**  **(Clobetasol propionate 0.05%)** | **VAS** |
| *Arduino, P. G. et al. (2018)* | 32 (NA) | NA | NA | 0, 2, 4, 6, 8 | 2 | ST  (Clobetasol propionate 0.05%) | Placebo | CS  VAS |
| *Georgaki, M. et al. (2022)* | 32 (71.87) | 61.08 | 59.60 | 0, 1, 2, 3, 4 | 3 | ST  (Dexamethason 2mg/5ml) | CNI  (Cyclosporin 100mg/ml) | CS  VAS |
| *Voute, A. B. E. et al. (1993)* | 40 (80.00) | NA | NA | 0, 3, 6, 9 | NA | ST  (Fluocinonide NA) | Placebo | VAS |
| *Volz, T. et al. (2008)* | 20 (75.00) | 59.40 | 59.40 | 0, 4 | 2 | CNI  (Pimecrolimus 1%) | Placebo | VAS |
| *Corrocher, G. et al. (2007)* | 32 (62.50) | 43.60 | 43.70 | 0, 4 | 4 | CNI  (Tacrolimus 0.1%) | ST  (Clobetasol propionate 0.05%) | Pain (0-3)  Burning sensation (0-3) |
| *Rödström, P. O. et al. (1994)* | 37 (NA) | NA | NA | 0, 3, 6, 9 | 3 | ST  (Clobetasol propionate 0.05%) | ST  (Triamcinolone acetonide 0.1%) | VAS  CS (0-3) |
| *Radfar, L. et al. (2007)* | 29 (55.17) | 59.00 | 58.00 | 0, 2, 6 | 2/1 based on response | CNI  (Tacrolimus 0.1%) | ST  (Clobetasol propionate 0.05%) | VAS  Lesion size |
| *Passeron, T. et al. (2007)* | 12 (NA) | NA | NA | 0, 4 | 2 | CNI  (Pimecrolimus 1%) | Placebo | Clinical score (0-12)  Score of erosions (0-4) |
| *Gorouhi, F. et al (2007)* | 40 (62.50) | 44.20 | 44.70 | 0, 4, 8 | 4 | CNI  (Pimecrolimus 1%) | ST  (Triamcinolone acetonide 0.1%) | CS  VAS  OHIP |
| *Azizi, A. et al. (2007)* | 60 (83.34) | NA | NA | 0, 4 | 4 | ST  (Triamcinolone acetonide 0.1%) | CNI  (Tacrolimus 0.1%) | CS  VAS |
| *Arduino, P. G. et al (2013)* | 30 (76.67) | 66.40 | 69.10 | 0, 2, 4, 6, 8 | 2 | CNI  (Tacrolimus 0.05%) | CNI  (Pimecrolimus 0.5%) | CS  VAS |
| *Sivaraman, S. et al. (2016)* | 30 (60.00) | 1: NA  2: NA | NA | 0, 1, 3, 6 | 4 | 1: ST  (Triamcinolone acetonide 0.1%)  2: ST  (Clobetasol propionate 0.05%) | CNI  (Tacrolimus 0.03%) | CS |
| *Siponen, M. et al. (2016)* | 27 (85.18) | 1: 60.00  2: 51.00 | 58.00 | 0, 3, 6 | 3 | 1: CNI  (Tacrolimus 0.1%)  2: ST  (Triamcinolone acetonide 0.1%) | Placebo | CS (0-130)  VAS |
| *Vohra, S. et al. (2015)* | 40 (47.50) | 39.80 | 31.70 | 0, 2, 4, 8 | 2 | CNI  (Tacrolimus 0.1%) | CNI  (Pimecrolimus 1%) | NCS  IL-6, IL-8 concentration |
| *Kaur, M. et al. (2016)* | 40 (65.00) | NA | NA | 0, 2, 4 | 3 | ST  (Clobetasol propionate 0.025%) | CNI  (Tacrolimus 0.1%) | CS  VAS |
| *Malik, S. et al. (2021)* | 80 (75.00) | 53.45 | 52.25 | 0, 2, 4, 8, 12 | 4 | CNI  (Tacrolimus 0.1%) | ST  (Triamcinolone acetonide NA) | Pain improvement |
| *Ibrahim, S. S. et al. (2023)* | 30 (90.00) | 1: 55.80  2: 48.60 | 41.10 | 0, 2, 4, 8 | 4 | 1: CNI  (Tacrolimus NA)  2: CNI  (Tacrolimus 0.1%) | ST  (Triamcinolone acetonide 0.1%) | CS  VAS  Total atrophic area |
| *McCaughey, C. et al. (2010)* | 21 (76.19) | 52.70 | 58.10 | 0, 6 | NA | CNI  (Pimecrolimus 1%) | Placebo | VAS  Erythema (0-3) |

**Supplementary table 1:** Basic Characteristics Table (bold letters represent included studies in the meta-analysis; n = 11) (CNI = Calcineurin inhibitor; ST = steroid; CS = Clinical score; VAS = Visual Analog Scale; NCS = Net Clinical Score)


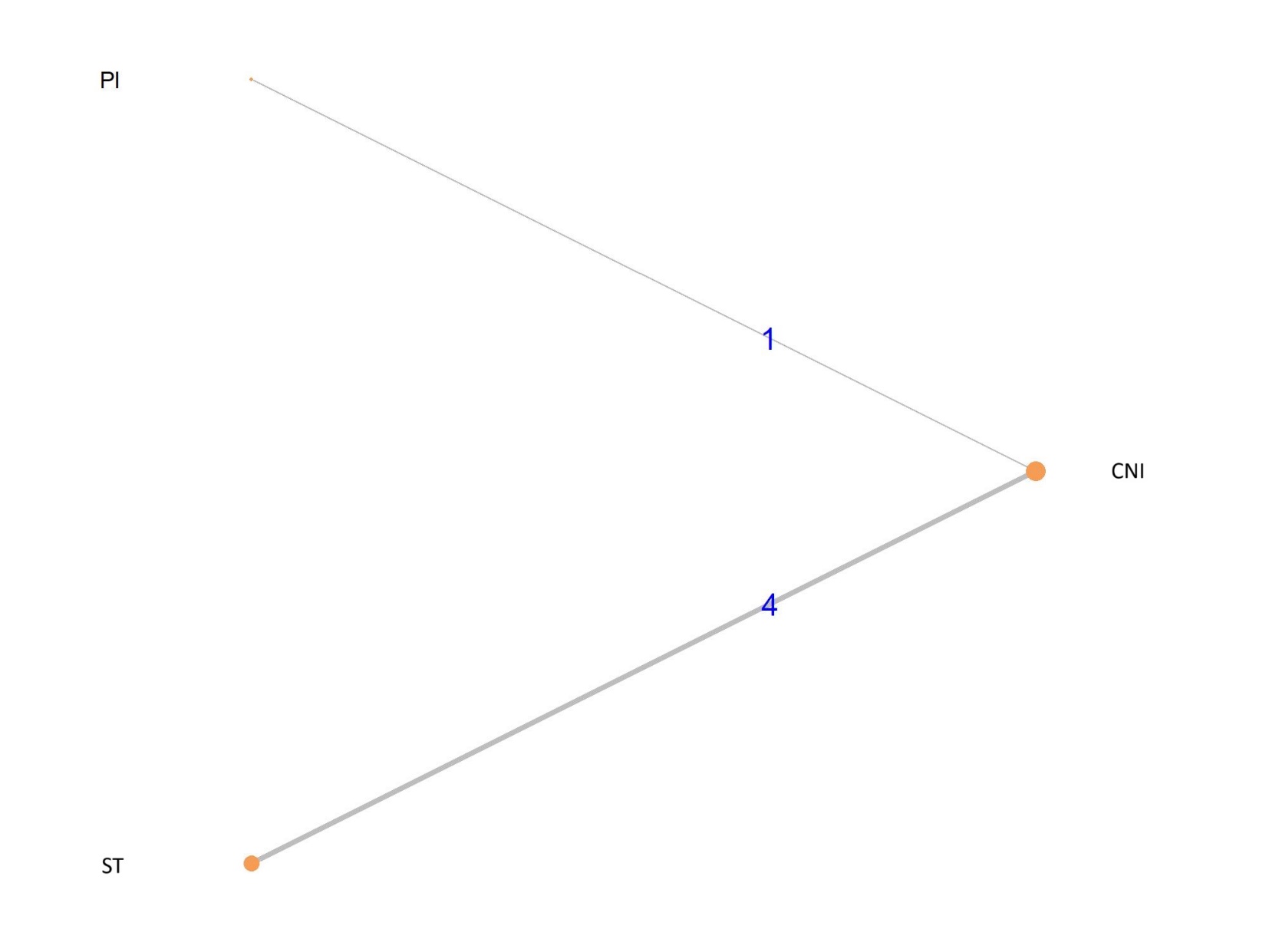


**Supplementary figure 1:** Network plot of included treatment regimens (steroid = ST, calcineurin inhibitors = CNI, placebo = Pl)

| **Score** | **Mucosal appearance** |
| --- | --- |
| *0* | *no lesion, normal mucosa* |
| *1* | *mild white striae, no erythematous area* |
| *2* | *white striae with an atrophic area less than 1* ${cm}^{2}$ |
| *3* | *white striae with atrophic area more than 1* ${cm}^{2}$ |
| *4* | *white striae with an erosive area less than 1* ${cm}^{2}$ |
| *5* | *white striae with an erosive area more than 1* ${cm}^{2}$ |

**Supplementary table 2:** Clinical Scoring System according to Thongprasom et al. (1)


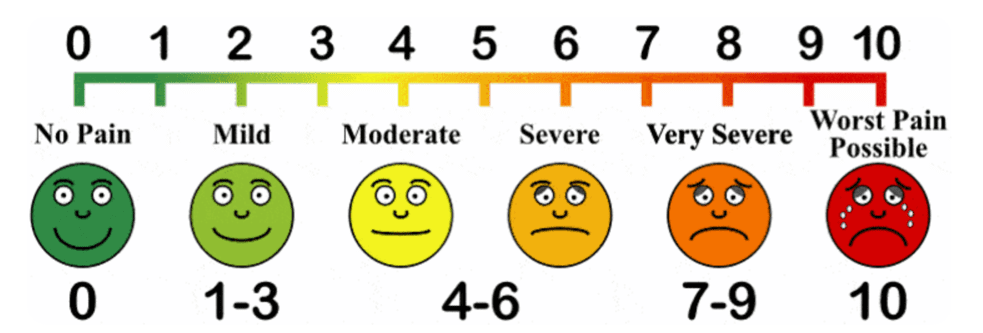


**Supplementary figure 2:** Visual Analog Scale (0-10 / 0-100) (2)


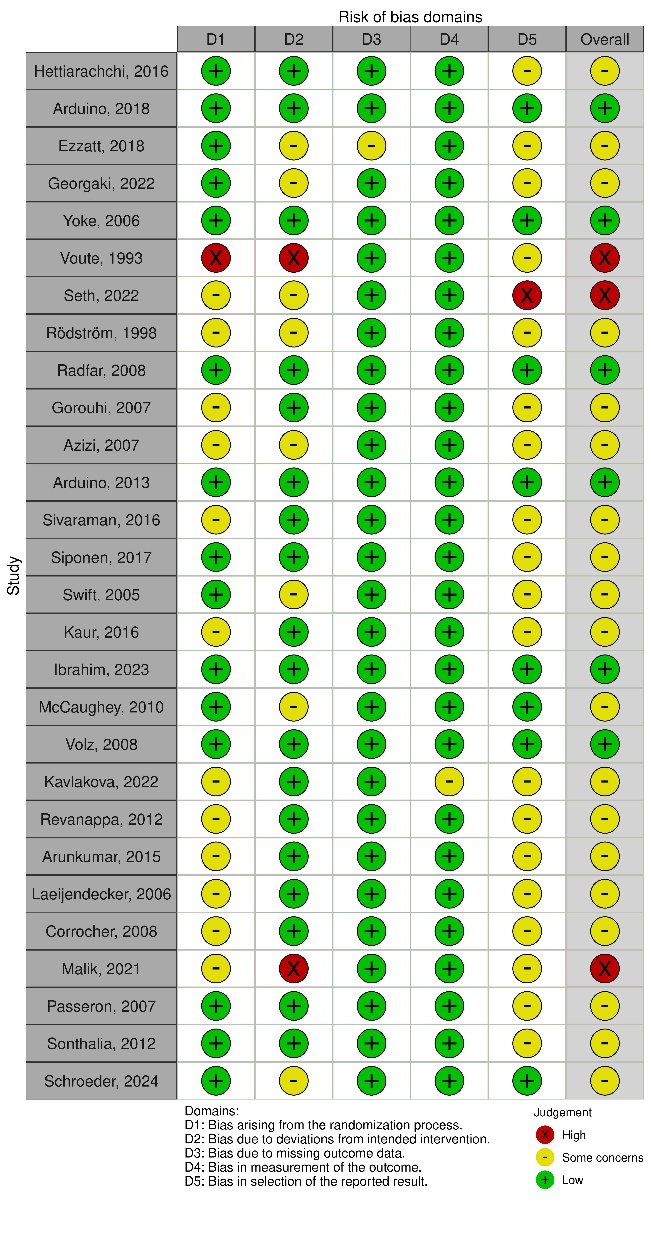

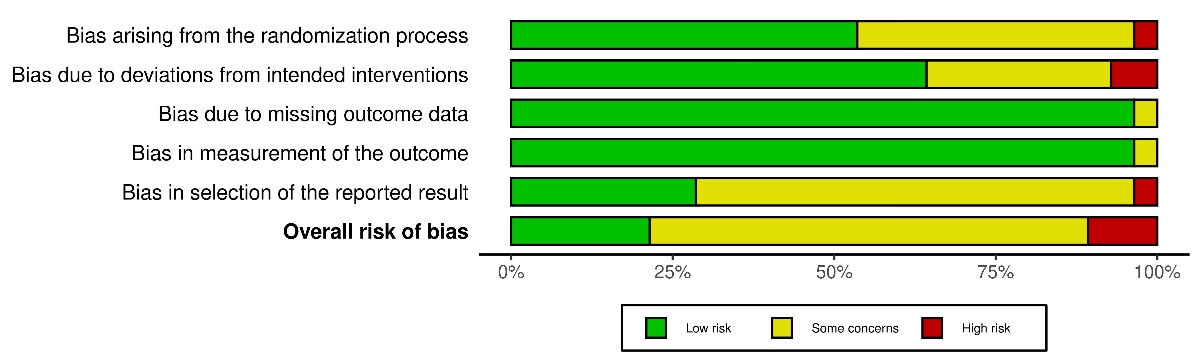


**Supplementary figure 3:** Risk of Bias Assessment with the use of the RoB 2 tool (3)


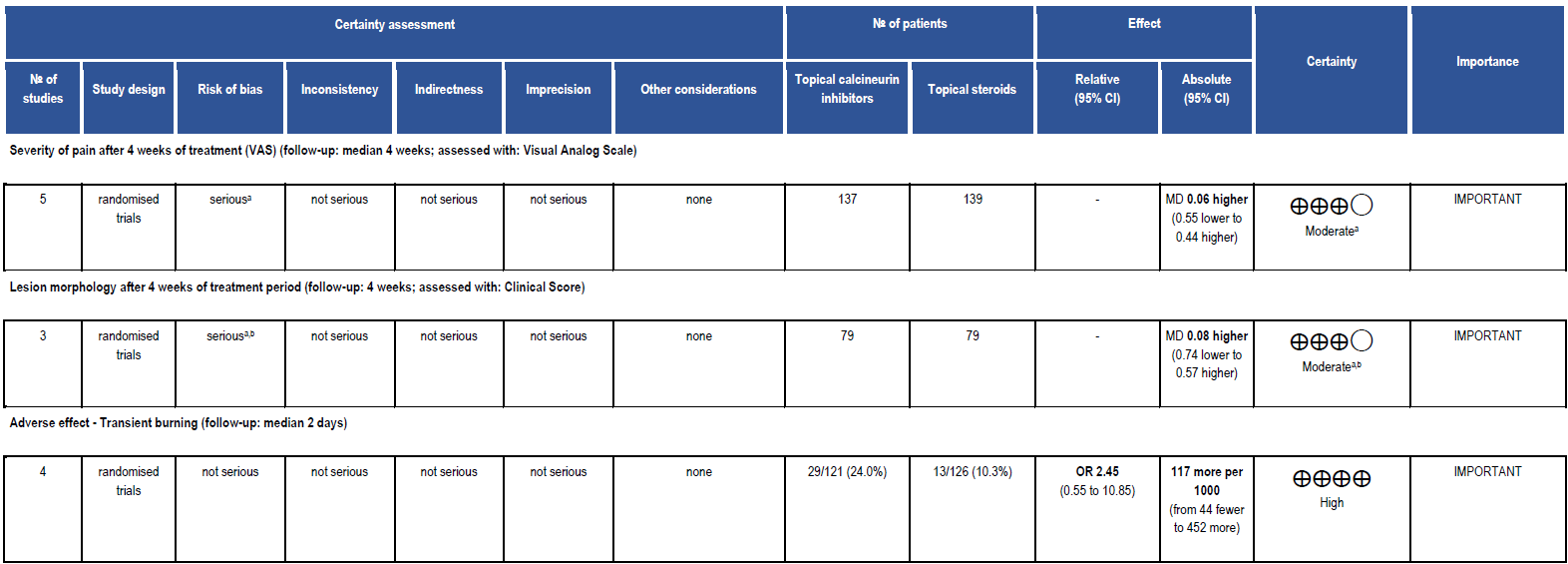


**Supplementary figure 4:** Quality of evidence assessment using GradePro tool (4)

1. Thongprasom K, Luangjarmekorn L, Sererat T, Taweesap W. Relative efficacy of fluocinolone acetonide compared with triamcinolone acetonide in treatment of oral lichen planus. J Oral Pathol Med. 1992;21(10):456-8.

2. Visual Analog Scale [Available from: <https://rebelem.com/haloperidol-for-treatment-of-headache-in-the-emergency-department/haldol-for-headache-vas-score/>.

3. Sterne JAC, Savović J, Page MJ, Elbers RG, Blencowe NS, Boutron I, et al. RoB 2: a revised tool for assessing risk of bias in randomised trials. Bmj. 2019;366:l4898.

4. Schünemann H BJ, Guyatt G, Oxman A, editors. GRADE handbook for grading quality of evidence and strength of recommendations. Updated October 2013: The GRADE Working Group, 2013; 2013.
